# Supplementary material for: A transcriptomic hourglass in brown algae
Source: Nature. 2024 Oct 23;635(8037):129–35. doi: 10.1038/s41586-024-08059-8 (PMC11540847; doi:10.1038/s41586-024-08059-8)
Supplement: Supplementary file 1 — Supplementary Tables 1–6 [file 41586_2024_8059_MOESM1_ESM.zip › 2024-03-05051B-s1/2024-03-05051B-SI-guide.docx]

# A transcriptomic hourglass in brown algae

Jaruwatana S. Lotharukpong^1^, Min Zheng^1^, Remy Luthringer^1^, Daniel Liesner^1^, Hajk-Georg Drost^2*^, Susana M. Coelho^1*^

^1^Department of Algal Development and Evolution, Max Planck Institute for Biology, Max-Planck-Ring 5, 72076 Tübingen, Germany

^2^Computational Biology Group, Department of Molecular Biology, Max Planck Institute for Biology, Max-Planck-Ring 5, 72076 Tübingen, Germany

*susana.coelho@tuebingen.mpg.de, hajk-georg.drost@tuebingen.mpg.de

**This compressed archival file (SupplementaryTables.tar.gz) contains Supplementary Tables 1-6.**

**Supplementary Table 1** – Sample list and sequencing data details including accessions, separated into two sheets. (1) Strains used in this study, (2) Further sample details.

**Supplementary Table 2** – Gene age and expression data per developmental stage (‘phyloexpression set’ format), separated into eight sheets. (1) *F. serratus*, (2) *F. distichus*, (3) *Ectocarpus* male strain EC32m, (4) *Ectocarpus* female strain EC25f, (5) *F. serratus* denoised, (6) *F. distichus* denoised, (7) *L. digitata*, (8) *S. polyschides*.

**Supplementary Table 3** – *tau* values per gene, separated into two sheets. (1) *F. serratus*, (2) *F. distichus*.

**Supplementary Table 4** – Permutation test statistics for (1) tissues in the mature SP of *F. serratus* and *F. distichus* and (2) *Ectocarpus* multicellular development.

**Supplementary Table 5** – dNdS values per gene in *Ectocarpus* (sp. 7), detailing the pairwise alignment match (reciprocal best hit) with *E. subulatus*, alignment scores, dN values, dS values and dNdS values.

**Supplementary Table 6** – GO terms from pTAI analysis, separated into four sheets. (1) *Ectocarpus* unicellular male (note: all non-significant), (2) *Ectocarpus* unicellular female (note: all non-significant), (3) *Ectocarpus* multicellular male, (4) *Ectocarpus* multicellular female.
